# Development of Highly Nutritional Breads with By-Products of Chia (*Salvia hispanica* L.) Seeds

**DOI:** 10.3390/foods9060819

**Published:** 2020-06-22

**Authors:** Estefanía Nancy Guiotto, Mabel Cristina Tomás, Claudia Mónika Haros

**Affiliations:** 1 Institute of Agrochemistry and Food Technology (IATA-CSIC), Av. Agustín Escardino 7 Parque Científico, 46980 Paterna-Valencia, Spain; esnagui@hotmail.com; 2 Centro de Investigación y Desarrollo, Criotecnología de Alimentos (CIDCA)—CCT La Plata-CONICET-Facultad de Ciencias Exactas (FCE) Universidad Nacional de La Plata (FCE-UNLP)—47 y 116, 1900, La Plata, Argentina; mabtom@hotmail.com

**Keywords:** *Salvia hispanica* L., residual chia flour, dough mixing behaviour, thermal properties, bread quality

## Abstract

The effect of the incorporation of various types of residual chia flour (whole, semi-defatted and defatted, with or without mucilage) on the technological quality of bread was investigated. The various types of chia flour were used to substitute 5 and 10% wt/wt of wheat flour in the bread formulations. The water absorption, dough development time and stability of blends with the presence of mucilage and the incorporation of 10% wt/wt of chia flour demonstrated the highest values in comparison with the other ones. The specific volume of the flour variants with 5% wt/wt of chia flour with mucilage were similar to the control bread; while those formulated with chia flour without mucilage exhibited a lesser volume. The incorporation of 10% wt/wt of chia flour in the formulations caused a decrease in the technological quality of the bread as expected. The bread crust and crumb colour parameters were mainly influenced by the level of chia flour substitution, which resulted in a decrease in lightness and h values. The substitution of wheat flour with 5% wt/wt of chia flour counterparts with mucilage improved the technological quality of the breads. The different oil content of the chia flours did not show any significant influence on overall quality or texture.

## 1. Introduction

Nowadays, consumers demand food products that have a high nutritional value and provide additional health benefits through the incorporation of new ingredients and natural products whose composition gives protective effects against certain diseases [1]. 

In this context, the incorporation of by-products derived from oilseeds, pseudocereals and other non-traditional crops could be of great interest for the development of new functional products [2].

Bread is a staple food, and in view of the high frequency of its consumption it can be considered a good delivery system for combining natural ingredients with nutritional and functional properties [3]. In recent years there has been great interest in optimizing bread-making technology to improve the quality and taste of the final products and the availability of the active compounds that they contain.

In this context, chia (*Salvia hispanica* L.) is an ancient seed that has been revalued in recent years because of its nutritional value [4]. Chia seeds are a good source of protein, dietary fibre and lipids, and over 60% of the total fatty acids are polyunsaturated fatty acids (PUFAs, omega-3) [5]. They are rich in essential amino acids, especially leucine, lysine, isoleucine and valine [6]. They also contain polyphenolic compounds such as caffeic, chlorogenic and rosmarinic acids and, to a lesser extent, quercetin, myricetin and kaempferol, which contribute to their high antioxidant capacity and low levels of lipid autoxidation products [7]. Moreover, chia seeds do not present anti-nutritional and vitamin-antagonistic factors like those found in other oilseeds such as flaxseed [5]. Another important characteristic of chia seeds is that they are gluten-free and can be consumed by people with celiac disease [4]. Furthermore, chia seeds and their by-products are particularly attractive because of their great water- and oil-holding capacity, characteristics that make them potential natural additives for baked goods [7].

The chia seed approval as a Novel Food by the European Parliament has led to a high degree of usage of chia seed in a wide range of foods at levels up to 10% [8,9,10]. There are several studies on the influence of chia on various food products, such as pasta [11], biscuits [12] and bread [13,14,15,16,17]. Recently, the use of two partially defatted powders of chia enriched with proteins or fibres was authorized as food supplements for the adult population, or as nutritional ingredients in a variety of foods [10].

Accordingly, the objective of this work was to develop bread incorporating different amounts of residual chia flours (whole, semi-defatted and defatted, with or without mucilage) as functional ingredients, and to evaluate the technological quality of the final bakery products.

## 2. Materials and Methods

### 2.1. Materials

Chia (*Salvia hispanica* L.) seeds were supplied by Nutracéutica Sturla S.R.L., Argentina. The seeds were cleaned manually and foreign matter, such as stones, dirt and broken seeds, was removed. Then they were packed in hermetic plastic vessels and stored at 5 °C until use. Before starting a test, the seeds were taken out of the refrigerator and allowed to temper at room temperature.

Commercial Spanish refined wheat flour was purchased from the local market. The flour alveograph parameters were tenacity, P: 60.3; extensibility, L: 64.0 mm; and deformation work, W: 151.7 × 10^−4^ J. Compressed yeast (*Saccharomyces cerevisiae*, Levital, Spain) was used as a starter for the breadmaking process. All the chemicals and solvents used were of analytical grade.

### 2.2. Preparation of Chia Flours

Whole chia flour (WCh) was obtained from black chia seeds ground in a laboratory grinder (Moulinex, horizontal blade grinder).

Semi-defatted chia flour with mucilage (SDCh) was obtained by cold pressing of chia seeds and further milling of the press-cake by the supplier (Nutracéutica Sturla S.R.L., Argentina).

Defatted chia flour with mucilage (DCh) was obtained from SDCh after oil extraction with n-hexane in a Soxhlet apparatus (Buenos Aires, Argentina) with thermal cycles at 80 °C, 8 h, following the IUPAC Standard Method [18].

The semi-defatted or defatted chia flours without mucilage (SDCh-OMu, DCh-OMu) were obtained from chia seeds after extraction of mucilage according to Capitani et al. [7] with modifications. Briefly, mucilage was extracted from whole chia seeds were soaked in water (1:10 wt/vt) for 1 h at room temperature with manual stirring. After 4 h, samples were frozen at −80 °C followed by freeze-drying (20 °C, 30 µm Hg, 72 h). The dried mucilage was separated from the nutlet by rubbing over a 20 ASTM mesh screen (840 μm). The oil content was extracted using a Soxhlet apparatus following the IUPAC Standard Method [18] with some modifications. The chia flours were homogenized and stored in plastic vessels at 5 ± 1 °C until subsequent use.

### 2.3. Determination of Flour Mixing Behaviour

A farinograph (Brabender, Duisburg, Germany) with a 300 g kneader was used to evaluate the impact of chia ingredients of the flour on the mixing behaviour, according to an Official Standard Method with minor modifications [19]. The thermostat was kept at 30 ± 2 °C and all the ingredients were mixed in the farinograph bowl to 500 BU (Brabender Units). The following parameters were determined in the farinograph analysis: Water absorption (WA) or percentage of water required to yield a dough consistency of 500 BU (Brabender Units); Dough development time (DDT), the time (min) taken to reach maximum consistency; Stability, the time during which dough consistency remained at 500 BU (min); Mixing tolerance index (MTI), the difference in consistency between height at peak and height 12 min later (BU); Farinograph quality number (FQN), is defined as the length from the point of the addition of water to the point 30 BU below the centre line of greatest consistency after the maximum along the time axis (mm). These features were read from paper records.

### 2.4. Breadmaking Procedure

The breads were prepared according to the formulation described by Iglesias-Puig and Haros [14] with some modifications. Various flour mixtures and two levels of substitution of wheat flour (5 and 10% wt/wt) were tested. The control dough formula consisted of wheat flour (300 g), compressed yeast (3% flour base), sodium salt (1.6% flour base) and distilled water (until optimum absorption, 500 BU). The ingredients were mixed for 5 min, allowed to stand for 10 min at high humidity, divided (75 g), rounded in a semi-automatic rounder and then allowed to ferment to an optimum volume increase at 28 ± 2 °C at 85% relative humidity. After the fermentation step, the doughs were baked in a laboratory electric oven with initial steaming (Salva, Lezo, Spain) at 215 °C for 17 min with six shaped dough pieces set on a baking plate. Then the buns were cooled to room temperature for 75 min for subsequent analysis. The experiments were performed in triplicate.

### 2.5. Proximate Composition

The ash and moisture of the flours were determined using AOCS procedure methods Ba 5a-49 and Ba 2a-38, respectively [20]. Protein content was calculated as nitrogen × 6.25 (AOAC Standard Method 920.87) [21] and total oil content was determined by extraction with hexane [18]. The dietary fibre content was measured by the total dietary fibre assay procedure [22]. Total carbohydrate content was estimated as nitrogen-free extract (NFE), which was calculated by difference using the equation:

NFE = 100 − (oil + protein + dietary fibre + ash)(1)

### 2.6. Technological Parameters

Loaf weight (g) and volume (cm^3^) were measured 1 h after removal from the oven. method [19]. Specific volume was calculated as the ratio of volume to weight (cm^3^/g), and the buns shape height/width ratio (or bun vaulting) of the central slice (cm/cm) was analysed. The images were acquired using a high Performance Full-HD Digital Microscope (EVO Cam II, Vision Engineering, Germany) with an image resolution of 16 megapixels, an identical settings of illumination was used for all images; then, they were analysed using the ImageJ/Fiji software (http://rsb.info.nih.gov/ij/) and the cells/cm^2^ were recorded [23]. 

Crust and crumb colours were evaluated by determining the tristimulus parameters L* (lightness), C* (chroma) and *h_ab_* (hue angle) of the baked loaves (crumb and crust) using a digital colorimeter (Chroma Meter CR-400, Konica Minolta Sensing, Japan), previously calibrated with the white plate supplied by the manufacturer. The instrument settings were as follows: illuminant D65, visual angle of 10° and calibration with Specular Component Included (SCI). From the parameters L* (lightness), a* (redness to greenness) and b* (yellowness to blueness) of the baked loaves (crumb and crust) the total colour difference Δ*E* was calculated by the equation:

Δ*E* = [(Δ*L*)^2^*+* (Δa*)^2^*+* (Δb*)^2^]^1/2^ (2)

Each determination was made in the centre of the bread by the extraction of five central slices from each sample; the evaluation was carried out in triplicate.

A Texture Profile Analysis (TPA) was performed, using a TA-XT2i texturometer (Stable Microsystems, Surrey, UK). Two 10-mm thick slices of each of three buns were compressed in the center of the Texture Analyzer platform using a cylindrical probe of 25 mm in diameter under the following conditions: speed of 1.7 mm/s for the test; speed of 10 mm/s for the post-test; 40% compression and 5 g trigger force. Firmness, Springiness, Cohesiveness, Resilience, Chewiness and Gumminess were recorded. The breads were then packed into polyethylene plastic bags and stored at room temperature (24 ± 2 °C) for the shelf life evaluation. The texture profile analysis was repeated after 1, 3 and 5 days of storage. Crumb slices with a thickness of 2 cm were compressed 50%. Four replicates from two different batches were analysed and averaged. The parameters recorded were hardness, chewiness, cohesiveness, springiness and resilience.

The flour’s thermal properties during baking of the fermented dough (gelatinization) and changes induced during storage of the bread (amylopectin retrogradation) were measured on a differential scanning calorimeter (DSC-7, PerkinElmer, Norwalk, CT, USA). Dough samples that were fermented (30–40 mg) were weighed directly in DSC stainless steel pans (LVC 0319-0218, PerkinElmer) and hermetically sealed (Quick-Press, 0990-8467, PerkinElmer, Norwalk, CT, USA). An empty pan was used as the reference. Briefly, the samples were kept at 30 °C for 1 min, heated from 30 to 110 °C at 11.7 °C/min, kept at this temperature for 5 min, and cooled to 30 °C at 50 °C/min, to simulate the temperature profile in the centre of the bread crumb during baking. A data analysis system (Mettler Toledo Star System) was used to determine the following parameters: Onset temperature (*To*), peak temperature (*Tp*), conclusion temperature (*Tc*) and enthalpy of gelatinization (Δ*H_G_*). To analyse amylopectin retrogradation, heated-cooled pans were stored at 20 °C for 15 days, and heated again in the calorimeter from 30 to 130 °C at 10 °C/min.

### 2.7. Statistical Analysis

Statistical analysis was performed by ANOVA at 5% significance level, followed by Tukey’s multiple comparison tests (*p* < 0.05). Data were processed using the Statgraphics Plus statistical package (Version 4.0 for Windows, Manugistics Inc., Rockville, MD, USA).

## 3. Results and Discussion

Proximate composition of the chia and wheat flours is shown in Table 1. As expected, whole chia flour (WCh) presented high protein and lipid contents, similarly to those found by other authors [13,16]. However, the dietary fibre content of this flour was higher than that reported by Fernandes et al. [24] (17.18 g/100 g). The difference in the composition of WCh can be attributed to differences in geographical origin, genotype and plant development stage, and to various production factors such as temperature, light and soil [24].

The protein content of the semi-defatted and fully defatted chia flours (with or without mucilage) was higher than that of the whole chia flour. The SDCh and DCh samples had total dietary fibre contents of 24.6 and 32.5%, respectively. These results were slightly higher than those of SDCh-OMu (22.6%) and DCh-OMu (30.3%), indicating the efficiency of the mucilage extraction. The defatted flours, DCh and DCh-OMu, had the highest dietary fibre and ash contents, with a significant decrease in lipid content (*p* < 0.05).

Cereal flours contain large quantities of starch, while chia seeds and their flours are virtually devoid of this carbohydrate. The presence of hydrophobic or water-binding components in a dough formulation might alter the mixing and overmixing properties of the hydrated flour, the thermal properties of starch and the bread quality [14].

**Figure 1:**
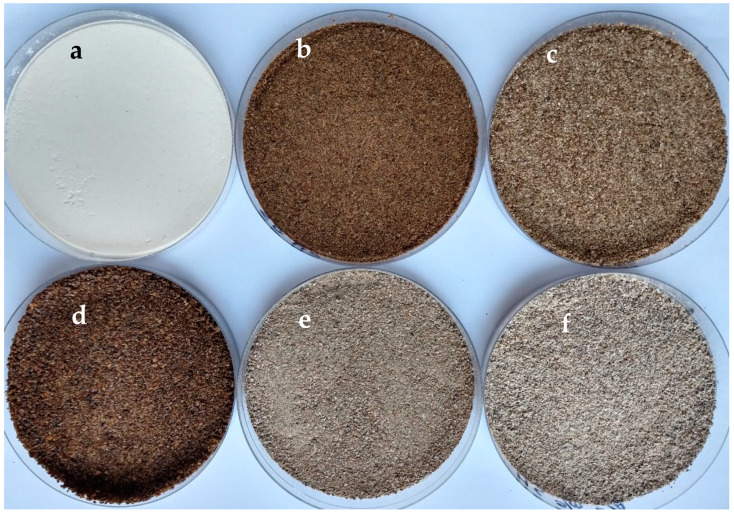
Raw materials: wheat flour (**a**); semi-defatted chia flour with mucilage (**b**); semi-defatted chia flour without mucilage (**c**); Whole chia flour (**d**); defatted chia flour with mucilage (**e**); defatted chia flour without mucilage (**f**).

The chia flour samples presented significant differences with respect to colour attributes such as L* (lightness), C* (Chroma), and *h_ab_* (hue) values. The extraction of oil and/or mucilage from chia flour increased L* and *h_ab_*, while the C* values decreased, and therefore DCh-OMu showed higher values for L* and *h_ab_*, and a lower value for C* (Figure 1).

Usually, a farinograph is used to evaluate the flour–water absorption required to reach a defined dough consistency, and to obtain the overall profile of dough during mixing/overmixing. The distribution of materials, hydration and energy input for stretching and alignment of protein molecules during mixing, involve shear and extensional deformation [14]. The farinographic properties of the doughs with different levels and types of chia flour are presented in Table 1. The water absorption (WA) of the SDCh-OMu and DCh-OMu doughs with a substitution of 5% wt/wt was similar to that recorded for the control system. The WA showed that the increase of chia flour level from 5 to 10% wt/wt produced a small increase in the absorption values of the various types of chia flour. This could be attributed to the increase in the dietary fibre content of the flour blends.

The inclusion of chia flour in the bread formulations had a considerable effect on the dough development time (DDT); when it was compared with the control system a significant increase was observed (*p* < 0.05). This effect could be related to the dilution of gluten and the difficulty of mixing fibre and wheat flour homogeneously. The DDT of the breads with SDCh or DCh was significantly prolonged than the breads with SDCh-OMu or DCh-OMu. This parameter was lowest in the case of bread with 5% wt/wt DCh-OMu (3.8 min) and highest in bread with 10% wt/wt SDCh (7.25 min). These results suggest that the modification of DDT was probably due mainly to the effect of the presence of chia mucilage in these systems. The stability of the dough containing chia flour without mucilage was comparable to that of the control dough, while the presence of mucilage in the dough produced an elevated stability, which was similar for both levels of substitution of chia flour. The control dough showed a remarkable higher mixing tolerance index (MTI) than those observed for the doughs with chia flour (*p* < 0.05). The different levels of chia flour substitution did not produce significant differences in MTI. However, significant differences between the various types of chia flour were recorded: WCh, SDCh and DCh had the lowest MTI values (*p* < 0.05).

The changes in this parameter due to the incorporation of chia flour may be attributed to the dilution of gluten-forming proteins, causing weakening of the dough. These results agree with those obtained by Koca and Anil [25], who reported that the incorporation of flaxseed in wheat flour produced a significant rise in water absorption and dough development time and a shortening of stability.

The FQN for the control dough was modified by chia flours addition (Table 1). This parameter was significantly higher for all the formulations with chia flour with mucilage, which indicated the improvement in the strength of this chia-wheat blends (*p* < 0.05). On the other hand, the substitution with chia ingredients without mucilage showed an increment in FQN but in a smaller proportion than the previous ones (*p* < 0.05). A high FQN means that a wheat flour is strong, it weakens late and slowly, whereas weak flour weakens early shown a low FQN [26]. The presence of the mucilage, even in lower proportion in the chia by-products without mucilage, seems to stabilize the interaction between the gluten proteins during the mixing step as was showed by the FQN parameter (Table 1).

The thermal properties of the samples are listed in Table 1. During the simulation of baking in the differential scanning calorimeter, the peak corresponding to the process of partial gelatinization of the amorphous phase of starch was observed between 61.1 and 76.6 °C, with a gelatinization enthalpy (Δ*H_G_*) (amount of energy required for this process) of 1.43 J/g of dough. The addition of 5% of WCh did not significantly alter the thermal properties, except to formulation with 5% of DCh which presented a significant increase on the temperatures and Δ*H_G_* (*p* < 0.05). The obtained results were according to those reported by Iglesias-Puig and Haros [14] who found that the thermal properties of the starch did not change substantially with the inclusion of chia ingredients at a 5 % level. On the other hand, the incorporation of 10% of different chia flours exhibited higher values on the parameters related to starch gelatinization (*To*, *Tp*) than the previous ones, except in those obtained with the addition of chia flours without mucilage (DCh-OMo, SDCh-OMo) (*p* < 0.05).

The chemical compositions of the bread samples containing different levels and types of chia flour are presented in Table 2. No significant differences were found in the moisture contents of the breads with the various substitution levels and types of chia flour in the systems. However, the proximate composition results showed that the incorporation of chia flour significantly increased the protein, dietary fibre and ash contents of the bread samples, while the carbohydrate content decreased in comparison with the control sample (*p* < 0.05). The contents of these components increased as the substitution levels of the various chia flours augment in the bread samples. The protein contents of the bread samples formulated with 5 or 10% wt/wt of semi-defatted or fully defatted chia flour had average values of 13.60 and 15.24%, respectively. Bread samples fortified with 10% DCh had the highest (7.62%) dietary fibre level.

When the breadmaking process ended, the shape, specific volume and pore size of the breads were evaluated and the results are presented in Table 2. It is noteworthy that the specific volume was affected by the level of wheat flour substitution. The incorporation of 10% wt/wt of the various types of chia flour caused low specific volumes. The specific volumes obtained for the breads of the experimental design varied from 2.59 to 3.99 mL/g (Table 2), with the highest volume corresponding to the incorporation of 5% wt/wt of WCh and the lowest one corresponding to 10% wt/wt of DCh-OMu. The incorporation of SDCh-OMu or DCh-OMu also caused a decrease in the specific volume, which could be due to the fact that chia flours have no gluten; moreover, the interactions between the proteins (gliadins and glutenins) of wheat flour and chia fibres can prevent the expansion of bread during the fermentation process [16]. This reduction in the volume was observed in other dough matrixes as in the cases of wheat flour enhancement by wholemeal from flaxseed, quinoa or amaranth [27,28,29]. Furthermore, the small volume level observed in the case of the incorporation of SDCh or DCh could be due to the low percentage of water in the formulation. Higher recipe portion of water allows easier fluffing up of the micropores in dough by fermentation gases, built up during mixing, then high amounts of water in formulations produce breads with high volumes [30]. 

**Figure 2:**
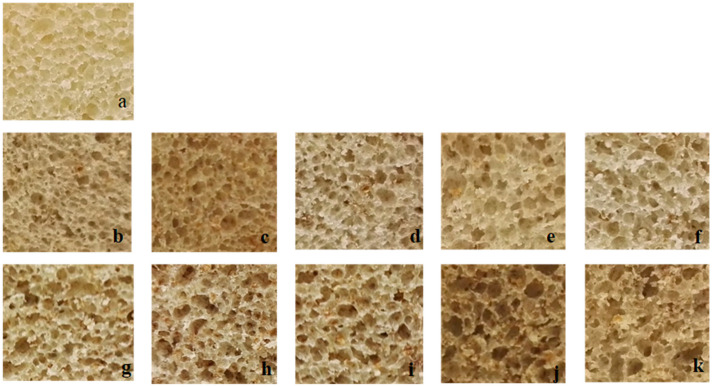
Effect of the incorporation of various types of chia flour on the central slice appearance. Bread formulation: Control (**a**); Whole chia flour 5% (**b**); semi-defatted chia flour with mucilage 5% (**c**); defatted chia flour with mucilage 5% (**d**); semi-defatted chia flour without mucilage 5% (**e**); defatted chia flour without mucilage 5% (**f**); Whole chia flour 10% (**g**); semi-defatted chia flour with mucilage 10% (**h**); defatted chia flour with mucilage 10% (**i**); semi-defatted chia flour without mucilage 10% (**j**); defatted chia flour without mucilage 10% (**k**).

The area of cells as a percentage of total slice area was used to describe crumb properties quantitatively. High values indicate a more open texture [31]. The microstructure of the loaves produced differed according to their composition (Table 2, Figure 2). 

The incorporation of 5% wt/wt of semi-defatted or fully defatted chia flour with or without mucilage (WCh, SDCh, SDCh-OMu, DCh or DCH-OMu) resulted in a large average pore size (Figure 2b–f). However, in these systems there was a great disparity in pore size. Small and large pores coexisted in the matrix, creating an uneven pore population. In contrast, the formulations with 10% wt/wt of chia flour had a more compact structure; the pores were smaller than those reported for other types of bread (Figure 2g–k). This result is in concordance with the bibliography take into account, that bread loaf volume is based on crumb texture—thickness of cell walls [32,33].

Table 2 shows the values of the colour parameters of crust and crumb for the samples of bread studied. These parameters were not influenced by the type of chia flour, indicating that the presence or absence of mucilage and the oil content did not contribute significantly to the colour values. However, the increase in the level of chia flour from 5 to 10% wt/wt resulted in a significant decrease in L* and *h_ab_* values (*p* < 0.05). These parameters showed a similar behaviour both in the crumb and in the crust of the bread samples. High L* values indicate high light reflectance, which suggests a light-coloured bread. The reduction of L* related to the incorporation of 10% wt/wt of chia flour could be due to the colour of this raw material. The chroma values (C*) of the crust decreased when the proportion of chia flour increased from 5 to 10%, while in crumb the C* values were not significantly affected by the incorporation of chia flour, irrespective of the amount and type incorporated. There were also significant differences in crust and crumb colour in comparison with the control, with values greater than 5 and therefore perceptible to the consumer, mainly with the incorporation of 10% of chia flour.

The texture parameters of the breads with different levels of substitution and types of chia flour are shown in Table 2. The hardness rose when the proportion of chia flour in the formulations increased. However, it must be emphasized that this parameter was lowered in the products with mucilage. According to Barcenas and Rosell [34], the inclusion of hydrocolloids in bread doughs improves the texture profile of the crumb, reducing its hardness.

**Figure 3:**
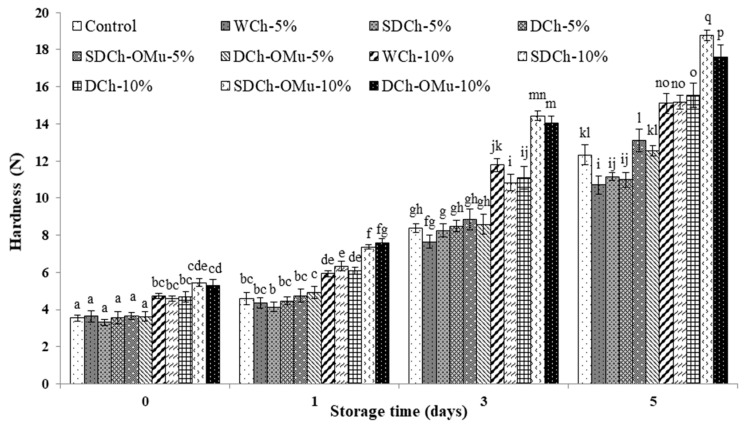
Hardness of breads formulated with different types and levels of chia flour during storage at 24 ± 2 °C. Values are the mean of three independent batches (n = 3) and vertical bars indicate standard deviation. WCh: Whole chia flour; SDCh: semi-defatted chia flour with mucilage; DCh: defatted chia flour with mucilage; SDCh-OMu: semi-defatted chia flour without mucilage; DCh-OMu: defatted chia flour without mucilage. ). Bars followed by different letters differ at *p* < 0.05, according to Tukey test.

The hardness of the loaves with 5% wt/wt of the various types of chia flour was not significantly different from that of the control bread, but the incorporation of 10% wt/wt of chia flour produced significant differences between the different breads. The highest hardness corresponded to the breads formulated with 10% wt/wt of SDCh-OMu or DCh-OMu. During storage, the hardness of all the samples analysed increased significantly (*p* < 0.05). The hardness of the loaves formulated with SDCh and DCh was significantly different, at both levels of substitution (5%, 10% wt/wt), from that of the other samples, indicating a positive effect on the crumb matrix (Figure 3). This could be due to the high water retention capacity associated with the presence of mucilage.

There were no significant differences in springiness between the samples of fresh bread or between samples during storage time (data not shown).

There was a significant decrease in cohesiveness when the level of chia flour increased, especially in the cases of SDCh and DCh, which could suggest that chia ingredients affect the strength of the internal bonds making up the crumb (the thickness and elasticity of the crumb cell walls) (*p* < 0.05).

Resilience, which is related to the instantaneous ability of crumb to recover its original geometry (elasticity), showed a similar behaviour to that of cohesiveness in the bread crumb. The inclusion of a higher amount of chia flour supported in chewiness and gumminess, and the presence of mucilage in the chia flour had a similar effect (Table 2).

The thermal properties of the samples are listed in Table 1. During the simulation of baking in the differential scanning calorimeter, the peak corresponding to the process of partial gelatinization of the amorphous phase of starch was observed between 61.1 and 76.6 °C, with a gelatinization enthalpy (Δ*H_G_*) (amount of energy required for this process) of 1.43 J/g of dough. The addition of 5% of WCh did not significantly alter the thermal properties, except to formulation with 5% of DCh which presented a significant increase on the temperatures and Δ*H_G_* (*p* < 0.05). The obtained results were according to those reported by Iglesias-Puig and Haros [14] who found that the thermal properties of the starch did not change substantially with the inclusion of chia ingredients at a 5 % level. On the other hand, the incorporation of 10% of different chia flours exhibited higher values on the parameters related to starch gelatinization (*To*, *Tp*) than the previous ones, except in those obtained with the addition of chia flours without mucilage (DCh-OMo, SDCh-OMo) (*p* < 0.05).

The evolution of amylopectin retrogradation is one of the main mechanisms involved in bread staling. After 15 days of storage at 20 °C, the retrogradation peak of the control system presented important changes respect to the initial time (t = 0), beginning at 40.4 °C and ending at 67.9 °C, with an enthalpy of 5.66 J/g of dough. A similar trend to the control bread was recorded after storage for the transition enthalpy and *To* values of the samples with different chia flours (data not shown). These results suggest that the addition of chia flours would not affect the bread staling. An opposite behaviour was observed by Iglesias-Puig and Haros [14], who found that the incorporation of chia inhibited the kinetics of amylopectin retrogradation during storage, which would be directly related to the delay in bread staling. 

## 4. Conclusions

This study shows that it is possible to make bread products with the characteristics of functional foods by adding chia seed by-products. The small incorporation of various types of chia flour in the formulations significantly increased the levels of proteins, dietary fibre and ash in the final products, compared with the wheat control. The presence of mucilage in the chia flours, at both levels studied, had a remarkable effect on the farinograph parameters evaluated, indicating higher water absorption, prolongation of dough development time and stability than in the case of the dough variants without mucilage.

The formulations containing 5% wt/wt of chia flour with mucilage (SDCh, DCh) presented the higher technological potential. However, the higher substitution (10% wt/wt) of wheat flour with the various types of chia flour affected the quality parameters, such as a decrease in the specific volume, an increase in the crumb hardness, and changes in the crumb and crust colour.

After the extraction of the oil and/or the mucilage of chia, the residual flours are produced, could have a nutritional and technologic potential application to be utilised not only in the cereal industry, but in the food industry in general.

## Figures and Tables

**Table 1:** Physico-chemical parameters of raw materials and dough formulations made with chia ingredients.

Parameter	Units	Wheat Flour	RAW MATERIALS—Chia By-Products									
			WCh		SDCh		DCh		SDCh-OMu		DCh-OMu	
***Proximate Composition***												
Moisture	% w.b.	5.5 ± 0.2 ^a^	7.0 ± 0.2 ^bc^		7.2 ± 0.1 ^c^		6.6 ± 0.2 ^b^		10.2 ± 0.2 ^e^		9.6 ± 0.2 ^d^	
Proteins	% d.b.	12.4 ± 0.2 ^a^	22.2 ± 0.7 ^b^		35.6 ± 0.6 ^c^		38.0 ± 0.5 ^d^		36.5 ± 0.3 ^c^		39.3 ± 0.6 ^d^	
Dietary Fibre	% d.b.	4.1 ± 0.8 ^a^	20.5 ± 0.8 ^b^		24.6 ± 0.2 ^d^		32.5 ± 0.7 ^f^		22.3 ± 0.3 ^c^		30.3 ± 0.6 ^e^	
Lipids	% d.b.	1.6 ± 0.2 ^b^	34.9 ± 0.2 ^d^		12.4 ± 0.4 ^c^		0.3 ± 0.3 ^a^		11.8 ± 0.2 ^c^		0.3 ± 0.1 ^a^	
NFE	% d.b.	81.26 ± 0.07 ^c^	16.82 ± 0.07 ^a^		21.23 ± 0.07 ^b^		21.07 ± 0.07 ^b^		22.5 ± 0.07 ^c^		21.60 ± 0.07 ^b^	
Ash	% d.b.	0.64 ± 0.03 ^a^	5.5 ± 0.3 ^b^		6.1 ± 0.5 ^c^		8.06 ± 0.08 ^d^		7.0 ± 0.1 ^c^		8.6 ± 0.2 ^e^	
***Colour***												
L*	–	93.11 ± 0.06 ^f^	30.71 ± 0.8 ^a^		38.7 ± 0.3 ^b^		56.2 ± 1.3 ^d^		51.2 ± 0.2 ^c^		66.8 ± 0.5 ^e^	
C*	–	9.21 ± 0.09 ^a^	24.7 ± 0.7 ^d^		19.7 ± 0.8 ^c^		10.9 ± 0.2 ^b^		18.0 ± 0.6 ^c^		10.9 ± 0.7 ^b^	
*h_ab_*	–	90.6 ± 0.1 ^d^	72.0 ± 0.3 ^a^		72.5 ± 0.9 ^a^		78.6 ± 0.3 ^b^		77.8 ± 0.7 ^b^		81.7 ± 0.2 ^c^	
**Parameter**	**Units**	**Control**	**DOUGH—Chia by-product level (% wt/wt)**									
			**WCh**		**SDCh**		**DCh**		**SDCh-OMu**		**DCh-OMu**	
			**5**	**10**	**5**	**10**	**5**	**10**	**5**	**10**	**5**	**10**
***Farinographic Properties***												
WA	%	58.1 ± 0.3 ^a^	59.7 ± 0.2 ^b^	61.2 ± 0.1 ^c^	60.0 ± 0.1 ^b^	63.1 ± 0.2 ^d^	61.10 ± 0.04 ^c^	64.4 ± 0.07 ^e^	58.4 ± 0.09 ^a^	59.9 ± 0.16 ^b^	58.1 ± 0.1 ^a^	59.6 ± 0.2 ^b^
DDT	min	1.90 ± 0.05 ^a^	5.00 ± 0.15 ^c^	6.00 ± 0.05 ^e^	5.80 ± 0.07 ^e^	7.25 ± 0.04 ^h^	6.25 ± 0.03 ^f^	6.90 ± 0.07 ^g^	4.05 ± 0.09 ^b^	5.45 ± 0.13 ^d^	3.8 ± 0.1 ^b^	5.3 ± 0.2 ^cd^
Stability	min	5.9 ± 0.2 ^a^	7.90 ± 0.05 ^c^	8.25 ± 0.05 ^c^	8.15 ± 0.05 ^c^	9.00 ± 0.05 ^d^	9.25 ± 0.05 ^d^	10.50 ± 0.05 ^e^	6.10 ± 0.08 ^ab^	6.35 ± 0.10 ^b^	5.8 ± 0.1 ^a^	6.05 ± 0.05 ^ab^
MTI	BU	150.0 ± 0.5 ^c^	105.0 ± 0.9 ^a^	100.0 ± 0.6 ^a^	100.0 ± 1 ^a^	100.0 ± 1 ^a^	100.0 ± 0.8 ^a^	105.0 ± 0.7 ^a^	130 ± 2 ^b^	135.0 ± 1.5 ^b^	140 ± 3 ^b^	135 ± 2 ^b^
FQN	mm	67 ± 2 ^a^	93 ± 4 ^cd^	105 ± 3 ^de^	102 ± 2 ^de^	123 ± 3 ^f^	108 ± 2 ^e^	126 ± 4 ^f^	81 ± 1 ^bc^	89 ± 2 ^c^	71 ± 1 ^ab^	84 ± 1 ^c^
***Thermal Properties***												
*To*	°C	61.1 ± 0.7 ^a^	61.5 ± 0.2 ^abc^	62.6 ± 1.0 ^e^	61.2 ± 0.1 ^ab^	61.9 ± 0.3 ^bcde^	62.1 ± 1.4 ^cde^	62.3 ± 0.2 ^de^	62.4 ± 0.6 ^de^	61.6 ± 0.1 ^abcd^	61.7 ± 0.1 ^abcd^	61.9 ± 0.8 ^abcde^
*Tp*	°C	68.7 ± 0.4 ^a^	69.5 ± 0.1 ^abc^	70.4 ± 1.1 ^d^	69.4 ± 0.0 ^ab^	69.9 ± 0.0 ^bcd^	70.3 ± 1.3 ^cdd^	70.4 ± 0.3 ^d^	69.7 ± 0.9 ^bcd^	69.5 ± 0.1 ^abc^	69.6 ± 0.3 ^bcd^	70.0 ± 0.8 ^bcd^
*Tc*	°C	76.6 ± 0.5 ^ab^	77.2 ± 0.3 ^a^	78.8 ± 1.7 ^cd^	80.4 ± 0.4 ^de^	80.0 ± 0.4 ^e^	80.1 ± 0.6 ^e^	80.2 ± 0.8 ^e^	78.0 ± 1.1 ^abc^	78.6 ± 0.0 ^bcd^	78.3 ± 0.1 ^a^^bcd^	80.4 ± 1.1 ^e^
Δ*H_G_*	J/g	1.43 ± 0.16 ^a^	1.5 ± 0.1 ^a^	1.58 ± 0.05 ^bc^	2.26 ± 0.02 ^g^	1.97 ± 0.01 ^ef^	1.90 ± 0.04^e^	1.68 ± 0.07 ^d^	1.40 ± 0.11 ^a^	1.67 ± 0.09 ^cd^	1.56 ± 0.01 ^b^	2.04 ± 0.03 ^f^

Mean values ± standard deviation (n = 3). Values followed by different letters differ at *p* ≤ 0.05, according to Tukey test. WCh: Whole chia flour; SDCh: semi-defatted chia flour with mucilage; DCh: defatted chia flour with mucilage; SDCh-OMu: semi-defatted chia flour without mucilage; DCh-OMu: defatted chia flour without mucilage; w.b.; wet basis¸d.b.: dry basis; wt/wt: weight/weight; WA: water absorption; DDT: Dough Development Time; MTI: Mixing Tolerance Index; FQN: Farinograph Quality Number; BU: Brabender Units; L*: lightness; C*: chroma; *h_ab_*: hue angle; *To*: onset temperature; *Tp*, peak temperature; *Tc*: conclusion temperature; Δ*H_G_*: enthalpy of gelatinization; J: Joules; NFE: nitrogen-free extract.

**Table 2:** Physico-chemical characteristics of fresh bread with chia by-products.

Bread Formula	Units	Control	Chia By-Products Level, % (wt/wt) on Flour Basis									
			WCh		SDCh		DCh		SDCh-OMu		DCh-OMu	
			5	10	5	10	5	10	5	10	5	10
**Proximate Composition**												
Moisture	% w.b	34.9 ± 0.5 ^a^	35.3 ± 0.5 ^a^	36.3 ± 0.6 ^a^	35.8 ± 0.7 ^a^	36.3 ± 0.5 ^a^	36.6 ± 0.7 ^a^	36.8 ± 0.5 ^a^	35.9 ± 0.4 ^a^	36.6 ± 0.6 ^a^	36.3 ± 0.8 ^a^	36.2 ± 0.4 ^a^
Proteins	% d.b	11.2 ± 0.5 ^a^	12.9 ± 0.7 ^ab^	13.6 ± 0.8 ^bc^	13.5 ± 0.3 ^bc^	14.9 ± 0.3 ^cd^	13.66 ± 0.07 ^bc^	15.25 ± 0.08 ^cd^	13.6 ± 0.3 ^bc^	15.0 ± 0.4 ^cd^	13.7 ± 0.6 ^bc^	15.8 ± 0.4 ^d^
Dietary Fibre	% d.b	2.64 ± 0.02 ^a^	4.88 ± 0.05 ^b^	5.7 ± 0.1 ^de^	5.09 ± 0.04 ^bc^	6.18 ± 0.08 ^f^	5.5 ± 0.1 ^d^	7.62 ± 0.09 ^h^	5.0 ± 0.1 ^b^	5.88 ± 0.09 ^ef^	5.4 ± 0.1 ^cd^	7.0 ± 0.1 ^g^
Lipids	% d.b	0.26 ± 0.03 ^a^	3.26 ± 0.07 ^e^	4.93 ± 0.09 ^f^	2.14 ± 0.03 ^c^	2.68 ± 0.03 ^d^	1.54 ± 0.06 ^b^	1.47 ± 0.07 ^b^	2.11 ± 0.08 ^c^	2.62 ± 0.05 ^d^	1.53 ± 0.04 ^b^	1.46 ± 0.02 ^b^
Carbohydrates	% d.b	85.7 ± 0.4 ^c^	78.1 ± 0.5 ^b^	74.7 ± 1.1 ^a^	78.3 ± 0.3 ^b^	75.1 ± 0.4 ^a^	78.30 ± 0.08 ^c^	74.3 ± 0.2 ^a^	78.4 ± 0.4 ^b^	75.2 ± 0.3 ^a^	78.3 ± 0.7 ^b^	74.4 ± 0.5 ^a^
Ash	% d.b	0.18 ± 0.02 ^a^	0.88 ± 0.05 ^b^	1.13 ± 0.06 ^cde^	0.91 ± 0.07 ^b^	1.19 ± 0.08 ^def^	1.01 ± 0.03 ^bcd^	1.38 ± 0.03 ^fg^	0.96 ± 0.05 ^bc^	1.27 ± 0.03 ^efg^	1.03 ± 0.08 ^bcd^	1.43 ± 0.04 ^g^
**Structural Characteristics**												
shape h/w ratio	cm/cm	0.58 ± 0.02 ^bc^	0.54 ± 0.02 ^ab^	0.66 ± 0.02 ^cd^	0.77 ± 0.04 ^f^	0.63 ± 0.01 ^c^	0.63 ± 0.02 ^c^	0.59 ± 0.03 ^bc^	0.68 ± 0.03 ^cd^	0.74 ± 0.02 ^ef^	0.75 ± 0.02 ^ef^	0.48 ± 0.01 ^a^
Specific Volume	mL/g	3.8 ± 0.1 ^d^	3.99 ± 0.08 ^d^	3.16 ± 0.07 ^bc^	3.8 ± 0.1 ^d^	2.97 ± 0.02 ^b^	3.97 ± 0.09 ^d^	3.03 ± 0.08 ^b^	3.32 ± 0.09 ^c^	2.7 ± 0.1 ^a^	3.24 ± 0.11 ^bc^	2.59 ± 0.07 ^a^
Cells/cm^2^	–	16.3 ± 0.4 ^ab^	14.8 ± 0.8 ^a^	18.6 ± 0.6 ^bc^	15.4 ± 0.5 ^a^	18.9 ± 0.7 ^bc^	15.2 ± 0.4 ^a^	19.4 ± 0.7 ^bc^	16.8 ± 0.3 ^b^	19.7 ± 0.8 ^c^	17.3 ± 0.6 ^b^	20.2 ± 0.4 ^c^
**Crust Colour**												
L*	–	63.8 ± 1.4 ^f^	55.6 ± 1.9 ^cd^	51.2 ± 0.9 ^b^	57.6 ± 0.6 ^e^	49.4 ± 2.1 ^a^	55.9 ± 1.4 ^cd^	50.8 ± 1.3 ^ab^	57.3 ± 0.9 ^e^	50.2 ± 1.0 ^a^	55.0 ± 1.6 ^c^	49.2 ± 1.8 ^a^
C*	–	35.3 ± 1.7 ^d^	33.7 ± 0.9 ^c^	32.0 ± 0.3 ^b^	34.8 ± 0.8 ^cd^	31.7 ± 0.9 ^b^	34.4 ± 0.5 ^cd^	32.3 ± 0.9 ^b^	33.6 ± 0.7 ^c^	30.7 ± 0.1 ^a^	33.8 ± 0.6 ^c^	30.37 ± 0.09 ^a^
*h_ab_*	–	76.6 ± 0.6 ^c^	73.9 ± 0.4 ^b^	71.1 ± 1.0 ª	73.8 ± 0.4 ^b^	70.9 ± 1.7 ª	73.5 ± 0.8 ^b^	71.0 ± 1.1 ª	73.0 ± 0.8 ^b^	70.4 ± 0.3 ª	73.7 ± 0.4 ^b^	70.5 ± 0.4 ^a^
Δ*E*	–	-	7.5 ± 1.1 ^a^	12.3 ± 1.4 ^b^	6.6 ± 1.2 ^a^	15.4 ± 1.1 ^b^	8.2 ± 0.9 ^a^	13.7 ± 0.9 ^b^	7.2 ± 1.6 ^a^	14.8 ± 1.5 ^b^	8.3 ± 0.8 ^a^	15.8 ± 0.7 ^b^
**Crumb Colour**												
L*	–	69.1 ± 2.0 ^c^	61.7 ± 2.0 ^b^	54.3 ± 1.5 ^a^	62.0 ± 1.2 ^b^	55.0 ± 0.3 ^a^	61.1 ± 0.9 ^b^	55.4 ± 1.6 ^a^	61.5 ± 1.7 ^b^	53.0 ± 1.5 ^a^	61.0 ± 0.3 ^b^	53.4 ± 1.2 ^a^
C*	–	14.2 ± 0.5 ^a^	14.7 ± 0.9 ^a^	14.7 ± 0.5 ^a^	14.1 ± 0.3 ^a^	14.0 ± 0.3 ^a^	14.0 ± 0.3 ^a^	15.0 ± 0.3 ^a^	13.7 ± 0.4 ^a^	13.2 ± 0.3 ^a^	13.6 ± 0.4 ^a^	13.7 ± 0.4 ^a^
*h_ab_*	–	96.8 ± 0.2 ^c^	90.9 ± 1.4 ^b^	85.2 ± 0.8 ^a^	89.8 ± 0.5 ^b^	85.9 ± 0.7 ^a^	88.3 ± 0.6 ^b^	84.2 ± 0.6 ^a^	90.0 ± 0.8 ^b^	84.7 ± 0.9 ^a^	89.4 ± 0.7 ^b^	85.3 ± 2.6 ^a^
Δ*E*	–	-	7.0 ± 0.9 ^a^	11.1 ± 1.0 ^c^	6.4 ± 0.8 ^a^	13.4 ± 1.2 ^d^	8.4 ± 1.1 ^b^	13.1 ± 1.3 ^d^	7.9 ± 0.9 ^ab^	16.4 ± 0.9 ^e^	9.3 ± 0.6 ^bc^	16.1 ± 1.0 ^e^
**Texture Parameters**												
Firmness	N	3.5 ± 0.2 ^a^	3.4 ± 0.3 ^a^	4.7 ± 0.2 ^b^	3.3 ± 0.1 ^a^	4.6 ± 0.2 ^b^	3.6 ± 0.2 ^a^	4.7 ± 0.3 ^b^	3.7 ± 0.2 ^a^	5.5 ± 0.3 ^c^	3.6 ± 0.3 ^a^	5.3 ± 0.2 ^c^
Springiness	mm	1.06 ± 0.02 ^a^	1.01 ± 0.02 ^a^	1.01 ± 0.02 ^a^	1.00 ± 0.01 ^a^	1.03 ± 0.01 ^a^	0.99 ± 0.02 ^a^	1.00 ± 0.02 ^a^	0.99 ± 0.02 ^a^	0.99 ± 0.02 ^a^	0.99 ± 0.01 ^a^	0.98 ± 0.01 ^a^
Cohesiveness	m/m	0.80 ± 0.02 ^b^	0.79 ± 0.02 ^b^	0.70 ± 0.06 ^ab^	0.80 ± 0.01 ^b^	0.68 ± 0.05 ^ab^	0.79 ± 0.02 ^b^	0.71 ± 0.04 ^ab^	0.74 ± 0.04 ^ab^	0.66 ± 0.06 ^a^	0.73 ± 0.03 ^ab^	0.69 ± 0.02 ^ab^
Resilience	N × mm	0.5 ± 0.1 ^b^	0.46 ± 0.03 ^b^	0.40 ± 0.06 ^ab^	0.45 ± 0.08 ^b^	0.4 ± 0.1 ^ab^	0.45 ± 0.06 ^b^	0.41 ± 0.08 ^ab^	0.4 ± 0.1 ^ab^	0.36 ± 0.09 ^a^	0.4 ± 0.1 ^ab^	0.4 ± 0.1 ^a^
Chewiness	N × mm	2.8 ± 0.1 ^a^	2.81 ± 0.05 ^a^	3.3 ± 0.1 ^ab^	2.6 ± 0.1 ^a^	3.4 ± 0.1 ^b^	2.74 ± 0.04 ^a^	3.37 ± 0.08 ^b^	2.96 ± 0.06 ^ab^	3.4 ± 0.2 ^b^	3.1 ± 0.2 ^ab^	3.4 ± 0.1 ^b^
Gumminess	N	2.64 ± 0.02 ^a^	3.00 ± 0.03 ^ab^	2.74 ± 0.03 ^a^	2.63 ± 0.02 ^a^	2.86 ± 0.06 ^ab^	2.94 ± 0.02 ^ab^	3.05 ± 0.05 ^ab^	3.09 ± 0.05 ^ab^	3.29 ± 0.04 ^b^	3.00 ± 0.06 ^ab^	3.38 ± 0.01 ^b^

Mean values ± standard deviation (n ≥ 3). Values followed by different letters differ at *p* < 0.05, according to Tukey test. WCh: Whole chia flour; SDCh: semi-defatted chia flour with mucilage; DCh: defatted chia flour with mucilage; SDCh-OMu: semi-defatted chia flour without mucilage; DCh-OMu: defatted chia flour without mucilage; h/w: hight/width; w.b.: wet basis; d.b.: dry basis; wt/wt: weight/weight; N: Newton; L*: lightness; C*: chroma; *h_ab_*: hue angle; Δ*E*: total colour difference, Δ*E =* [(Δ*L*)^2^ + (Δa*)^2^ + (Δb*)^2^]^1/2^; a*: redness to greenness; b*: yellowness to blueness.
